# Association Between Derivatives of Reactive Oxygen Metabolites and Hemodynamics in Children with Left-to-Right Shunt Congenital Heart Disease

**DOI:** 10.3390/antiox13111294

**Published:** 2024-10-25

**Authors:** Takamichi Ishikawa, Daisuke Masui, Hiroki Uchiyama

**Affiliations:** Department of Pediatrics, Hamamatsu University School of Medicine, 1-20-1 Handayama, Chuo-ku, Hamamatsu 431-3192, Japan; 41248479@hama-med.ac.jp (D.M.); 07485516@hama-med.ac.jp (H.U.)

**Keywords:** oxidative stress, congenital heart disease, reactive oxygen metabolites, children

## Abstract

Existing reports on the association between oxidative stress and pulmonary hemodynamics in congenital heart disease (CHD) are limited, and the relationship remains inadequately understood. To address this, we evaluated the link between oxidative stress and hemodynamics in children with left-to-right shunt CHD. We analyzed the derivatives of reactive oxygen metabolites (d-ROMs) in a cohort of 60 children with left-to-right shunt CHD and compared them to 60 healthy, age- and sex-matched controls. In the CHD group, hemodynamics measured by cardiac catheterization were evaluated in relation to d-ROMs. We also assessed the diagnostic performance of the d-ROMs for a pulmonary-to-systemic blood flow ratio (Qp/Qs) of >1.5. We found that the blood d-ROM levels in the CHD group were significantly higher than those in the control group (*p* < 0.001). A significant positive correlation was observed between d-ROMs and Qp/Qs (*p* < 0.001), d-ROMs and the ratio of the right ventricular end-diastolic volume (*p* < 0.001), d-ROMs and the mean pulmonary arterial pressure (*p* < 0.001), and d-ROMs and the ratio of the left ventricular end-diastolic volume (*p* = 0.007). In the receiver operating characteristic curve analysis, the area under the curve for d-ROMs in predicting Qp/Qs > 1.5 was 0.806 (*p* < 0.001), which, although not statistically significant, was higher than that of the plasma N-terminal pro-brain natriuretic peptide (0.716). These findings indicate that d-ROM levels are closely associated with hemodynamics and the disease severity in patients with left-to-right shunt CHD and may serve as a valuable marker for determining the need for surgical intervention.

## 1. Introduction

Congenital heart disease (CHD) represents the most common congenital anomaly and remains the leading cause of morbidity and mortality in children. This condition encompasses a broad spectrum of hemodynamic abnormalities caused by structural impairments that occur during the fetal developmental stage of the heart, as well as various associated diseases. The severity of CHD is highly variable, ranging from cases that resolve spontaneously to those necessitating surgical intervention in the neonatal period. A thorough understanding of the pathophysiology of CHD is crucial for the effective management of these patients and is key to improving their prognosis.

Oxidative stress arises when the production of reactive oxygen species (ROS), which play a crucial role in maintaining redox homeostasis, surpasses the body’s capacity to effectively neutralize and eliminate them. This disruption in the balance between ROS generation and removal leads to a state of oxidative imbalance, which is increasingly being recognized as a central contributor to the pathophysiology of numerous diseases. In the context of cardiovascular health, oxidative stress is closely linked to myocardial remodeling, a process that significantly affects the structure and function of the heart. Additionally, elevated oxidative stress levels have been identified as key predictors of heart failure severity and are associated with increased cardiovascular mortality in the adult population [1]. The derivatives of reactive oxygen metabolites (d-ROM) test is a method used to quantify the hydroperoxides formed through the peroxidation of alkoxy and peroxy-radicals, using a chromogen-based coloring solution. This test measures hydroperoxide levels as an indicator of ROS, providing a straightforward and direct assessment of the total oxidative status [2,3,4]. The application of the d-ROM test offers significant advantages, particularly owing to its efficiency and ease of use in clinical diagnostics. A key benefit of this method is its minimal sample requirement, needing only 10–20 μL of serum or plasma, which reduces the invasiveness of the procedure. Additionally, the test employs a simplified protocol, wherein the addition and mixing of a color-developing chromogen solution constitutes the entire preparatory process. This streamlined approach is further enhanced by the rapidity of the photometric analysis, which can be completed in approximately 5 minutes using a dedicated measuring device [2,3]. This emerging biomarker has been utilized to evaluate disease severity and therapeutic efficacy in cardiovascular conditions, including heart failure, coronary artery disease, and atrial fibrillation [5].

Recent studies have explored the relationship between oxidative stress and various pediatric diseases [6,7]. However, the association between oxidative stress and pulmonary hemodynamics in CHD remains limited and not well understood [8,9,10]. This study aimed to investigate the role of oxidative stress in children with left-to-right shunt CHD and to determine whether d-ROMs are a suitable prognostic marker for assessing the severity of left-to-right shunt CHD, potentially serving as an indicator for the timing of surgical intervention.

## 2. Materials and Methods

### 2.1. Study Population

We prospectively enrolled 60 patients diagnosed with left-to-right shunt CHD and 60 healthy controls matched for age and sex. All patients were diagnosed with left-to-right shunt CHD and underwent cardiac catheterization at Hamamatsu University Hospital in Japan. Based on sex and date of birth (with a tolerance of ±1 year), a 1:1 match of children who underwent a health checkup with a normal physical examination and did not have evidence of disease history was utilized as the control group. Patients with conditions associated with the formation of free radicals were excluded as follows: pulmonary disease, hypoxia, septicemia, other congenital anomalies, ecchymosis, metabolic disease, polycythemia, cephalohematoma, systemic disease, and a family history of smoking. The subjects were recruited throughout the study period from April 2018 to March 2023. The study was conducted in accordance with the ethical principles outlined in the Declaration of Helsinki and received approval from the Hamamatsu University School of Medicine (protocol no. 18-029, on 26 April 2018). Informed consent was obtained from all parents or legal guardians, who provided written approval for the children’s participation before their enrollment in the study.

### 2.2. Definitions of CHD

CHD was defined as a significant structural abnormality of the heart or intrathoracic great vessels, either associated with functional impairment or possessing potential clinical significance [11]. Given the frequent occurrence of patent ductus arteriosus (PDA) in pediatric CHD cases, infants with PDA were included in this study only if they were born after 37 weeks of gestation and the PDA failed to close spontaneously within the first month of life [12]. An atrial septal defect (ASD) was defined as the presence of an interatrial communication measuring ≥4 mm in diameter, accompanied by the dilation of the right atrium and right ventricle. In contrast, an intra-atrial defect measuring <4 mm at the fossa ovalis was classified as a patent foramen ovale [13].

### 2.3. Evaluation of CHD and Hemodynamics

All patients were diagnosed with left-to-right shunt CHD by echocardiography using either PHILIPS iE33 or Affiniti 50 (Philips Medical Systems, Andover, MA, USA) ultrasound devices with a 5 or 8 MHz transducer. The examination protocol incorporated 2-dimensional and color Doppler imaging, performed systematically from the parasternal, suprasternal, subxiphoid, and apical views. Modified views were employed to ensure comprehensive diagnostic accuracy when standard views were insufficient for optimal visualization. To enhance the precision of the imaging and reduce the likelihood of false-positive signals, special care was taken to adjust the color-flow mapping settings. Specifically, the gain and filter parameters were meticulously calibrated to minimize artifacts and ensure the reliability of the Doppler signals [14,15]. Each echocardiographic examination was performed by one of the two pediatric cardiologists (T. I. and H. U.). The pulmonary-to-systemic blood flow ratio (Qp/Qs) was calculated using the Fick method to assess hemodynamics, based on data obtained from cardiac catheterization. Blood samples were collected to measure the venous and arterial oxygen saturation (SO_2_), which is a crucial step in the calculation of Qp/Qs. Additionally, right and left ventriculograms were performed to evaluate the right and left ventricular end-diastolic volumes (RVEDV and LVEDV, respectively). RVEDV was calculated using the Simpson method [16] based on right ventriculography, while LVEDV was determined using the area–length method [17] based on left ventriculography. The ratio of the end-diastolic right (%RVEDV) and left (%LVEDV) ventricular volumes under normal values was calculated using Nakazawa’s normal value [18].

### 2.4. Serum Measurements

The ROS levels were assessed using a kinetic spectrophotometric assay with a Free Radical Elective Analyzer (FREE^®^; Wismerll Co., Ltd., Tokyo, Japan). The principles underlying the d-ROM test have been previously elucidated [2,3]. The d-ROM test employs spectrophotometry to detect the oxidation of N, N-diethyl-para-phenylenediamine, a chromogenic substrate, by radicals derived from hydroperoxide. To measure d-ROM levels, 20 μL of blood was added to 1 mL of buffer in a cuvette, which was then gently inverted up and down for 10 s to mix. Following this, 20 μL of chromogenic substrate was introduced into the cuvette. After thorough mixing, the cuvette was placed in a thermostatically controlled space with a photometer, and optical measurements were taken at 505 nm for 5 min [19]. The test results are reported in Carratelli Units (U.CARR), with 1 U.CARR being equivalent to 0.08 mg of H_2_O_2_ per 100 mL. The plasma levels of N-terminal pro-brain natriuretic peptide (NT-proBNP) were determined using an Elecsys 2010 analyzer and a chemiluminescent immunoassay kit (Roche Diagnostics GmbH, Mannheim, Germany).

### 2.5. Statistical Analyses

Continuous variables were presented as the mean ± standard deviation or as the median with interquartile range (IQR), depending on the distribution of the data. Baseline characteristics between the two groups were compared using the two-sided Student’s t-test for normally distributed data, whereas the Mann–Whitney U test was applied for skewed data. Qualitative categorical variables were analyzed using the chi-square test or Fisher’s exact test, depending on the sample size and expected frequencies. Spearman’s correlation coefficient was used to examine the relationships between the d-ROM levels and hemodynamic parameters, given its robustness in handling non-parametric data. The predictive capacity of the biomarker levels to distinguish Qp/Qs > 1.5 was assessed through a receiver operating characteristic (ROC) curve analysis. This analysis used the calculation of the area under the curve (AUC), along with specificity and sensitivity, to evaluate the diagnostic performance. A *p*-value of less than 0.05 was considered indicative of statistical significance across all analyses. All statistical analyses were performed using SPSS version 29.0 (IBM Corp., Armonk, NY, USA).

## 3. Results

The median age of all participants was 7.0 months (IQR of 3.0 to 61.0). Among the 60 children diagnosed with CHD, the majority (*n* = 41) presented with ventricular septal defect (VSD), followed by 12 patients with ASD, 4 patients with atrioventricular septal defect (AVSD), and 3 patients with PDA. The baseline and biological characteristics of the CHD and control groups are summarized in Table 1. No statistically significant differences were observed between the two groups, which ensured comparability. The laboratory and hemodynamic parameters specific to the CHD group are shown in Table 2.

**Figure 1:**
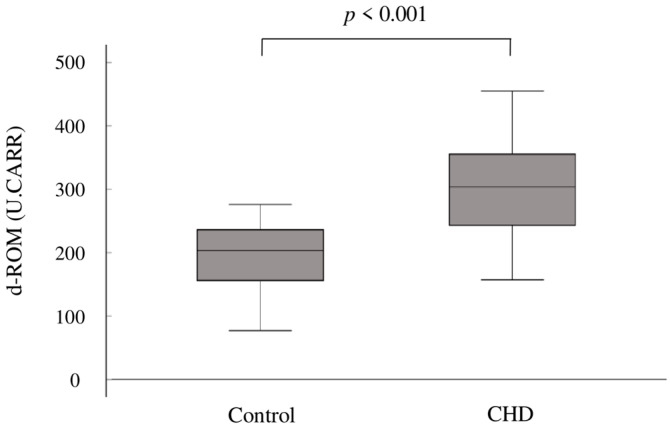
Box plots showing the distribution of d-ROMs in the two groups. The upper boundary of the box represents the 75th percentile, and the lower boundary of the box represents the 25th percentile. The line through each box represents the median value in each group. d-ROMs, derivatives of reactive oxygen metabolites; CHD, congenital heart disease.

In terms of oxidative stress, the mean d-ROM levels were significantly elevated in the CHD group compared to the control group (302 ± 75 U.CARR vs. 199 ± 46 U.CARR; *p* < 0.001) (Figure 1).

**Figure 2:**
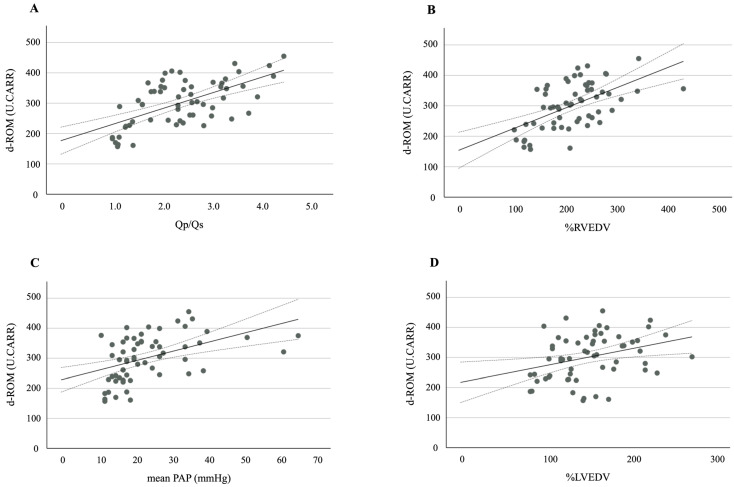
A scatter plot and correlation between d-ROM and (**A**) Qp/Qs, (**B**) %RVEDV, (**C**) mean PAP, and (**D**) %LVEDV. The solid line represents the regression line. The dotted lines show the 95% confidence interval. d-ROMs, derivatives of reactive oxygen metabolites; Qp/Qs, pulmonary-to-systemic blood flow ratio; %RVEDV, ratio of right ventricular end-diastolic volume; PAP, pulmonary arterial pressure; %LVEDV, ratio of left ventricular end-diastolic volume.

A further analysis revealed strong positive correlations between the d-ROM levels and the following hemodynamic variables: Qp/Qs (r = 0.607, *p* < 0.001), %RVEDV (r = 0.542, *p* < 0.001), mean PAP (r = 0.474, *p* < 0.001), and %LVEDV (r = 0.346, *p* = 0.007) (Figure 2A–D). A significant positive correlation was also observed between the d-ROM and NT-proBNP levels (r = 0.399, *p* = 0.002).

**Figure 3:**
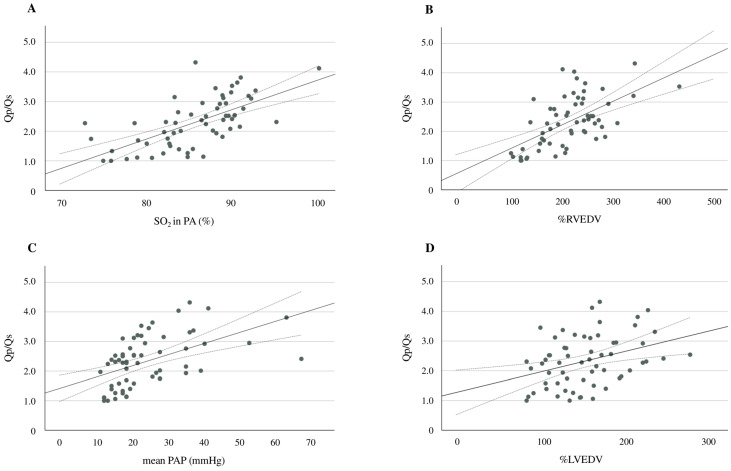
A scatter plot and the correlation between d-ROMs and pulmonary arterial SO_2_ (**A**), Qp/Qs and %RVEDV (**B**), Qp/Qs and the mean PAP (**C**), and Qp/Qs and %LVEDV (**D**). The solid line represents the regression line. The dotted lines show the 95% confidence interval. d-ROMs, derivatives of reactive oxygen metabolites; SO_2_, oxygen saturation; Qp/Qs, pulmonary-to-systemic blood flow ratio; %RVEDV, ratio of right ventricular end-diastolic volume; PAP, pulmonary arterial pressure; %LVEDV, ratio of left ventricular end-diastolic volume.

**Figure 4:**
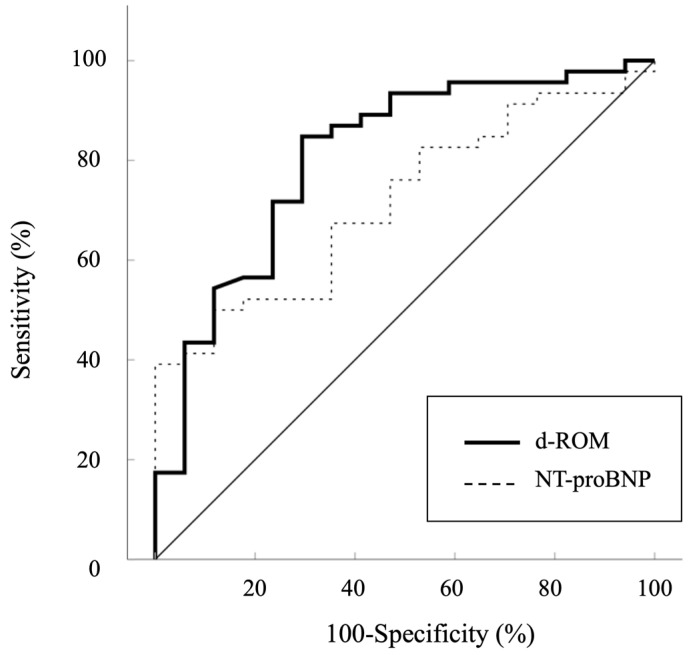
Receiver operator curves analysis used to determine the diagnostic performance of d-ROMs for Qp/Qs > 1.5. d-ROMs, derivatives of reactive oxygen metabolites; NT-proBNP, plasma N-terminal pro-brain natriuretic peptide.

Additional analyses showed significant positive correlations between Qp/Qs and pulmonary arterial SO_2_ (r = 0.720, *p* < 0.001), Qp/Qs and %RVEDV (r = 0.594, *p* < 0.001), Qp/Qs and mean PAP (r = 0.510, *p* < 0.001), and Qp/Qs and %LVEDV (r = 0.359, *p* = 0.004) (Figure 3A–D). An ROC curve analysis was performed to evaluate the predictive ability of d-ROMs in comparison to NT-proBNP for identifying Qp/Qs > 1.5. The AUC for d-ROMs was 0.806 (95% CI: 0.680–0.932, *p* < 0.001), while the AUC for NT-proBNP was 0.716 (95% CI: 0.587–0.846, *p* = 0.001) (Figure 4). Although d-ROMs exhibited a higher AUC value, the comparison of AUC values between d-ROMs and NT-proBNP did not reach statistical significance (*p* = 0.307). The optimal cut-off value for d-ROMs, determined by the ROC curve, was 253 U.CARR, yielding a sensitivity of 0.848 and a specificity of 0.706.

## 4. Discussion

In comparison to previous studies that explored the relationship between oxidative stress and left-to-right shunt CHD, our study stands out due to its larger sample size and the inclusion of the youngest participants [8,9,10,20]. Notably, while earlier studies primarily relied on echocardiography to evaluate hemodynamics, we employed cardiac catheterization in all patients with CHD, allowing for a more precise and accurate assessment of hemodynamic parameters. The key findings of our study are as follows. First, there was a significant elevation in d-ROM levels, an established marker of oxidative stress, among children with left-to-right shunt CHD compared to the control group. Second, we observed a significant positive correlation between d-ROM levels and several critical hemodynamic parameters (Qp/Qs, %RVEDV, mean PAP, and %LVEDV). Finally, although statistical significance was not reached, the AUC value for d-ROMs in predicting Qp/Qs > 1.5 was higher than that of NT-proBNP, suggesting the potential utility of d-ROMs as a predictive marker. These findings underscore the relevance of oxidative stress in the pathophysiology of left-to-right shunt CHD and highlight the possible role of d-ROM in future diagnostic and prognostic assessments.

The current study found that the serum d-ROM levels were significantly elevated in patients with left-to-right shunt CHD compared to the controls. This elevated d-ROM level indicates oxidative stress and free radical-mediated damage, which may contribute to detrimental changes in myocardial cells, including reduced myocardial function. The primary hemodynamic etiologies are volume overload in ASD, and are volume and pressure overload in VSD, PDA, and AVSD. The cardiac catheterization results suggest that the high d-ROM levels are attributed to these overload conditions. Volume and pressure overloads induce distinct patterns of cardiac remodeling. Pressure overload typically results in concentric hypertrophy, while volume overload is associated with eccentric hypertrophy. Both types of overloads generate mechanical stress, which lead to myocardial stretching and remodeling. The mechanical stretching of cardiomyocytes increases ROS production, which has been linked to hypertrophic cardiac remodeling and apoptosis [21,22]. Increased ROS production limits the bioavailability of nitric oxide in neighboring cardiomyocytes, which subsequently reduces protein kinase G (PKG) activity. Diminished PKG activity removes the inhibition of cardiomyocyte hypertrophy, thereby promoting concentric left ventricular remodeling. Additionally, it contributes to cardiomyocyte stiffening due to the hypophosphorylation of the giant cytoskeletal protein, titin. A combination of stiffened cardiomyocytes and increased collagen deposition by myofibroblasts results in ventricular dysfunction. These processes ultimately contribute to the progression of heart failure [23,24].

In this study, Qp/Qs showed the highest correlation with d-ROMs among the parameters evaluated. The Qp/Qs ratio is a critical parameter for assessing hemodynamics in CHD, and a value of 1.5 or greater is considered an indication for surgical intervention. Although this did not reach statistical significance, the AUC value for Qp/Qs > 1.5 for d-ROMs was higher than that for NT-proBNP. Furthermore, a significant positive correlation was observed between the d-ROM and NT-proBNP levels. BNP is a cardiac hormone known for its diverse physiological roles, with natriuretic, vasodilatory, diuretic, and antifibrotic effects. It is widely recognized as a key biomarker for assessing both right and left ventricular function and for diagnosing heart failure [25]. BNP is primarily secreted by ventricular myocytes in response to increased ventricular wall stress, which often results from a volume overload due to a significant left-to-right shunt. Recent research has strengthened the hypothesis that natriuretic peptides play a crucial role in detecting the early signs of ventricular dysfunction in patients with CHD [26,27]. Previous studies have consistently demonstrated significant positive correlations between plasma BNP levels and the Qp/Qs ratio, a measure that reflects the extent of the CHD-related shunt and the corresponding volume overload imposed on the right ventricle and pulmonary artery [28,29]. These findings highlight the utility of BNP as a marker for evaluating the severity of the volume load in patients with CHD. Considering these observations, our study extends this understanding by proposing that d-ROMs, an oxidative stress marker, can also indicate pressure and volume loads in the right ventricle and pulmonary artery, serving as an additional tool for assessing the severity of left-to-right shunt CHD. In the current study, we observed that %RVEDV and the mean PAP exhibited stronger correlations with d-ROM levels than %LVEDV. These findings may be explained by the physiological mechanisms previously outlined, wherein right ventricular and pulmonary artery pressure and volume overload play a dominant role in the generation of oxidative stress. Our findings indicate that d-ROMs may offer a diagnostic value equivalent to or greater than NT-proBNP for determining the need for surgical intervention in children with left-to-right shunt CHD.

Pulmonary arterial hypertension (PAH) associated with CHD represents a severe and potentially life-threatening condition that can develop in children because of excessive pulmonary blood flow. In our study, we observed a significant correlation between elevated levels of d-ROMs, a marker of oxidative stress, and increased pulmonary arterial pressure in pediatric patients with CHD. This elevation in oxidative stress appears to be closely associated with endothelial dysfunction in the pulmonary vasculature, which is a hallmark of PAH. Specifically, damage to vascular endothelial cells in PAH contributes to impaired nitric oxide synthase activity within the pulmonary endothelium. As the pulmonary arterial pressure continues to rise, the degree of endothelial cell damage intensifies, further promoting ROS overproduction [30]. Therefore, oxidative stress plays a pivotal role in the pathophysiology of PAH. Previous studies have identified multiple sources of ROS that exacerbate oxidative stress and contribute to subsequent vascular dysfunction. For example, in a neonatal lamb model of left-to-right shunt CHD, secondary pulmonary hypertension induced by increased pulmonary blood flow was strongly linked to enhanced oxidative stress [31]. In another study, plasma malondialdehyde levels, a well-established biomarker of ROS, were significantly elevated in PAH patients [32]. Zhang et al. demonstrated a positive correlation between serum asymmetric dimethylarginine (ADMA) levels and the mean PAP in patients with PAH. ADMA, a naturally occurring amino acid, is an endogenous inhibitor of nitric oxide synthase. The lungs serve as a primary source of both nitric oxide synthase and ADMA, which are maintained in a dynamic balance that regulates nitric oxide production. Their findings suggest that the downregulation of the nitric oxide/cGMP pathway plays a pivotal role in the pathogenesis of PAH, and the extent of inhibition within this pathway directly influences disease severity [33]. These substances contribute to endothelial dysfunction, increased vascular tone, and blood vessel remodeling.

In this study, we did not include patients with cyanotic CHD. Previous reports have indicated that cyanotic CHD exhibits higher levels of oxidative stress than acyanotic CHD [34]. Ercan et al. demonstrated that oxidative stress markers, including the total oxidative status, total antioxidant status, and oxidative stress index, are significantly elevated in patients with cyanotic heart disease compared to healthy controls [8]. Cyanotic CHD leads to chronic hypoxia, a state in which oxygen deficiency depletes the body’s antioxidant reserves, thereby increasing susceptibility to oxidative stress. This oxidative imbalance is believed to exacerbate lipid peroxidation, further damaging cell membranes and contributing to the pathophysiology of cyanotic CHD. Accelerated lipid peroxidation in these patients may also have downstream effects on vascular function and overall cardiovascular health. In a previous study, a significant negative correlation was observed between oxygen saturation and oxidative stress markers in patients with cyanotic CHD, suggesting that the degree of hypoxemia directly influences oxidative damage. Lower oxygen saturation levels are associated with higher oxidative stress, underscoring the critical role that oxygen deficiency plays in increasing oxidative vulnerability in this patient population [9]. In contrast, the present study focused on patients with left-to-right shunt CHD who were not cyanotic. As these patients did not experience chronic oxygen deficiency, it is plausible that a distinct mechanism underlies the observed increase in oxidative stress. This study identified a significant positive correlation between d-ROM levels and pulmonary arterial oxygen saturation, suggesting that beyond compensatory mechanisms, elevated pulmonary arterial oxygenation may also contribute to increased oxidative stress in this patient population. Surgical intervention is required for all children diagnosed with cyanotic CHD due to the severity of the condition. Conversely, not all patients with acyanotic CHD, especially those with left-to-right shunt CHD, require surgical treatment. Given the variability in the need for surgery among patients with acyanotic CHD, it is of paramount importance to establish reliable methods to accurately determine which individuals will benefit from surgical intervention. The ability to differentiate between patients who require surgery and those who can be managed conservatively is critical for optimizing patient outcomes. In this context, the discovery and validation of easily accessible biomarkers that can assist in making clinical decisions are of great significance. Such biomarkers would provide clinicians with a non-invasive, cost-effective tool for determining the necessity of surgical treatment and guiding the timing in patients with left-to-right shunt CHD. Given that d-ROMs require only a minimal blood sample and can be measured both quickly and easily, they hold significant potential for widespread application in clinical practice, particularly in pediatric care, including for newborns and infants. Our findings suggest that d-ROM levels may serve as a noninvasive marker for determining the need for surgical intervention. A d-ROM level greater than 253 U.CARR indicates a Qp/Qs ratio exceeding 1.5 and could be used to guide surgical decisions in children with left-to-right shunt CHD. Furthermore, the cutoff value, when used in conjunction with BNP and NT-proBNP, may provide a more accurate prediction for surgical intervention. However, long-term follow-up studies are necessary to further validate the effectiveness of d-ROM levels in monitoring patient health and their implications for optimizing CHD management strategies.

This study had several limitations that must be acknowledged. First, the sample size was relatively modest, consisting of 60 children with CHD and 60 control subjects. Although this represents one of the largest studies of its kind, the statistical power may still be insufficient to establish robust correlations between the d-ROM levels and various hemodynamic parameters. A larger cohort is necessary to enhance statistical rigor and further validate the findings. Second, while this study focused on oxidative stress associated with left-to-right shunt, it is important to consider the potential impact of oxidative stress during the fetal period, which may also contribute to the onset and progression of CHD. Although genetic susceptibility plays a pivotal role in the pathogenesis of many forms of CHD, a range of environmental factors, including maternal alcohol consumption, cigarette smoking, exposure to industrial chemicals, and viral infections, are also thought to be critically involved. These environmental factors are believed to exert embryotoxic effects primarily through the excessive production of ROS and the subsequent impairment of the body’s antioxidant defense mechanisms. This imbalance, characterized by elevated ROS levels and diminished antioxidant capacity, has profound implications for cardiac organogenesis. In addition to its effect on organ development, oxidative stress may have teratogenic consequences by altering gene expression levels during embryonic development. Such disruptions can result in the death of cardiac neural crest cells, significantly increasing the risk of CHD, particularly in cases involving conotruncal and outflow tract abnormalities [35]. In the present study, it is not possible to rule out the possibility that the elevated ROS levels observed in the CHD group may have been influenced by oxidative stress during fetal development, in addition to the volume and pressure overload associated with left-to-right shunts.

## 5. Conclusions

In conclusion, our study confirmed the presence of oxidative stress in children with CHD and established a significant association with left-to-right shunts. This represents the first investigation to explore the potential use of d-ROMs as a biomarker in the context of CHD. These findings suggest that oxidative stress may play a key role in the progression of left-to-right shunt CHD, providing a novel avenue for both diagnostic and therapeutic approaches. Although large-scale studies are necessary to further substantiate the role of d-ROMs and to elucidate their full clinical utility, our research offers preliminary evidence to suggest that this biomarker could serve as a valuable tool for assessing disease severity. Moreover, the d-ROM levels may provide critical insights into the timing of surgical intervention, potentially improving patient outcomes by guiding more precise and timely treatment decisions. Future research, particularly at the molecular level, could further clarify the underlying pathophysiological mechanisms linking oxidative stress with CHD, ultimately contributing to the development of targeted therapeutic strategies.

## Figures and Tables

**Table 1:** Clinical characteristics of the study group.

	Control (*n* = 60)	CHD (*n* = 60)	*p* Value
Age (months)	6.0 (3.0–48.8)	8.5 (2.8–53.5)	0.710
Sex (male/female)	30/30	30/30	1.000
Height (cm)	70.4 (60.2–108.6)	68.8 (57.6–105.3)	0.558
Weight (kg)	7.6 (5.8–17.0)	7.4 (4.8–16.4)	0.707
BMI (kg/m^2^)	15.7 (14.4–17.1)	14.8 (14.1–16.4)	0.496
HR (beats/min)	118 (93–124)	120 (104–130)	0.191
Systolic BP (mm Hg)	82 (78–95)	81 (76–90)	0.177
Diastolic BP (mm Hg)	47 (42–56)	46.0 (41–52)	0.165

Values are expressed as the median (interquartile range). BMI, body mass index; HR, heart rate; BP, blood pressure.

**Table 2:** Laboratory and hemodynamic characteristics of the CHD group.

	CHD (*n* = 60)
SO_2_ in aorta (%)	97.8 ± 2.3
SO_2_ in PA (%)	85.6 ± 5.4
Qp/Qs	2.33 ± 0.86
mPAP (mmHg)	24 ± 11
%RVEDV	176 ± 50
%LVEDV	152 ± 44
d-ROM (U.CARR)	302 ± 75
NT-proBNP (pg/mL)	380 * (98–1066)

Values are expressed as mean ± standard deviation. * Values are expressed as the median (interquartile range). SO_2_, oxygen saturation; PA, pulmonary artery; Qp/Qs, pulmonary-to-systemic blood flow ratio; PAP, pulmonary arterial pressure; RVEDV, right ventricular end-diastolic volume; LVEDV, left ventricular end-diastolic volume; ROMs, reactive oxygen metabolites; NT-proBNP, N-terminal pro-brain natriuretic peptide.

## Data Availability

The data presented in this study are available upon reasonable request to the corresponding author. The data were not publicly available due to ethical considerations.
